# Verification of Official LC-MS/MS Methods and Nationwide Monitoring of Five Prohibited Veterinary Drugs and a Major Metabolite in Livestock Muscle in Korea

**DOI:** 10.3390/foods15203662

**Published:** 2026-10-15

**Authors:** Sunjin Park, Se-Hyung Kim, Chung-Oui Hong, Do Hui Kim, Seon-Young Lee, Soo Kyoung Lee, Seok-Jae Kim, Mi-Young Park

**Affiliations:** 1 Livestock Product Export and Sanitation Team, Animal and Plant Quarantine Agency (APQA), 177, Hyeoksin 8-ro, Gimcheon-si 39660, Gyeongsangbuk-do, Republic of Korea; bluedonald@korea.kr (S.P.);; 2 Department of Companion Animal Health Care, Kyung-In Woman’s University, 63, Gyeyangsan-ro, Gyeyang-gu, Incheon 21041, Republic of Korea

**Keywords:** prohibited veterinary drugs, livestock muscle, LC-MS/MS, analytical performance verification, nationwide monitoring, food safety

## Abstract

This study evaluated the fitness for purpose of official Korean Food Code LC-MS/MS methods for five prohibited veterinary drugs and one major metabolite—pyrimethamine, dapsone, monoacetyldapsone, roxarsone, chloramphenicol, and vancomycin—in bovine, porcine, and chicken muscle and applied the evaluated methods to nationwide monitoring of production-stage samples. In 2024, dapsone, monoacetyldapsone, pyrimethamine, and roxarsone were analyzed in 202 muscle samples, whereas in 2025, chloramphenicol and vancomycin were analyzed in 300 muscle samples (total *n* = 502). Analytical performance was assessed for selectivity, linearity, limits of detection (LOD) and quantification (LOQ), recovery, repeatability, inter-day precision, and between-analyst performance; all analyte–matrix combinations met the predefined acceptance criteria. For chloramphenicol, an LOQ of 0.15 μg/kg was achieved in all three matrices, equal to the European Union reference point for action. An external proficiency test for chloramphenicol in bovine muscle yielded a satisfactory z-score of −0.30. No sample met the regulatory reporting criterion for any target analyte included in the respective annual survey. These findings support the fitness for purpose of the evaluated official methods and provide baseline analytical and occurrence data for surveillance of selected prohibited substances in production-stage meat in Korea.

## 1. Introduction

Veterinary medicinal products and feed additives have long been used in animal production to prevent and treat diseases and to improve productivity [1]. However, residues of administered compounds or their metabolites may remain in edible animal tissues, resulting in consumer exposure through food. Depending on the properties of the compound, such exposure may raise public health concerns, including toxicity, genotoxicity, and antimicrobial resistance [2,3]. Consequently, regulatory authorities establish maximum residue limits for authorized veterinary drugs, while certain substances associated with substantial safety concerns or not authorized for use in food-producing animals are separately designated as substances that must not be detected in food. Because regulatory enforcement for these substances requires selective identification and reliable quantification at trace concentrations, the analytical performance of official methods can directly affect the effectiveness of residue control.

Major regulatory jurisdictions, including the European Union [4], the United States [5], Canada [6], Australia [7], and China [8], regulate pharmacologically active substances that are prohibited, unauthorized, or otherwise restricted for use in food-producing animals through their respective legislation and control lists. In Korea, a regulatory list of veterinary drugs that must not be detected in food was introduced in 2007 [9]. The scope of this list has subsequently been expanded to reflect the results of risk assessments and developments in international regulations. The current Korean Food Code designates a total of 30 substances as substances that must not be detected in food (Table 1 and Table S1). These include chloramphenicol, nitrofuran and nitroimidazole compounds, and various other antimicrobials, growth promoters, and pharmacologically active substances [9].

The Korean National Residue Program (NRP) is a nationwide surveillance system designed to monitor residues of veterinary drugs, pesticides, and environmental contaminants in livestock products at the production stage. However, not all substances designated in the Korean Food Code as substances that must not be detected in food are included in routine annual testing. In addition to routine monitoring, Animal and Plant Quarantine Agency (APQA) conducts targeted annual surveillance of selected prohibited substances, with a limited number of analytes prioritized each year according to monitoring needs. The present study was conducted within this targeted surveillance framework and was not intended to comprehensively evaluate all prohibited substances listed in the Korean Food Code; rather, it focused on the analytes selected for the 2024 and 2025 annual targeted surveillance programs. Accordingly, the study was designed as a focused evaluation of the fitness for purpose of the relevant official Korean Food Code methods in bovine, porcine, and chicken muscle, followed by their application to production-stage monitoring.

The analytes selected for the present study comprised five prohibited parent substances—pyrimethamine (PYR), dapsone (DAP), roxarsone (ROX), chloramphenicol (CAP), and vancomycin (VCM)—together with monoacetyldapsone (MAD), a major metabolite of DAP. PYR was formerly used as an anticoccidial agent in poultry [10,11,12,13], and hematopoietic abnormalities and developmental toxicity have been reported following high-dose or prolonged exposure [14,15]. DAP is a diphenylsulfone antibacterial agent associated with hematotoxicity [16,17]. Because DAP is metabolized in vivo to MAD, both the parent compound and its metabolite should be considered in residue assessment. ROX is an organoarsenical compound that was formerly used in poultry production for growth promotion and coccidiosis control [18]. Concerns have been raised because ROX can be transformed into other organoarsenic species or inorganic arsenic in animals and the environment [19,20,21,22].

CAP is a well-recognized prohibited antimicrobial associated with serious adverse effects, including aplastic anemia [2,23]. VCM is a glycopeptide antimicrobial that plays an important role in the treatment of severe infections caused by Gram-positive bacteria [24,25,26]. The 2024 World Health Organization (WHO) List of Medically Important Antimicrobials classifies glycopeptides and lipoglycopeptides, including VCM, as ‘authorized for use in humans only’ [27]. WHO further notes that antimicrobial classes in this category should be regarded as having the highest level of concern for antimicrobial-resistance risk and should not be newly authorized for use in food-producing animals. This classification underscores the importance of preserving the therapeutic effectiveness of glycopeptide antimicrobials in human medicine [27].

The limit of quantification (LOQ) specified for CAP in the official Korean Food Code method is 0.5 µg/kg [28], whereas the European Union has established a reference point for action (RPA) of 0.15 µg/kg for CAP, a pharmacologically active substance not authorized for use in food-producing animals [29]. Analytical capability at the EU RPA is therefore relevant when comparing method sensitivity with EU regulatory control levels and may also be useful for residue surveillance associated with international trade. Although CAP is already included in routine NRP testing, it was included in the present study as part of the 2025 targeted surveillance program, with additional emphasis on evaluating the performance of the official method at the EU RPA. In contrast, DAP, PYR, ROX, and VCM, although regulated in Korea as substances that must not be detected in food, are not currently included among the routine testing items of the NRP. Consequently, nationwide information on their occurrence in domestically produced meat remains limited.

Accordingly, this study aimed to evaluate the species-specific fitness for purpose of the official Korean Food Code LC-MS/MS methods for PYR, DAP, MAD, ROX, CAP, and VCM in bovine, porcine, and chicken muscle and to apply the evaluated methods to production-stage monitoring in Korea. By integrating analytical performance verification with monitoring data, the study sought to provide baseline analytical and occurrence information for the selected target analytes, particularly those for which routine domestic monitoring data are limited.

## 2. Materials and Methods

### 2.1. Reagents and Standard Solutions

Analytical standards of DAP, MAD, PYR, ROX, CAP, and VCM, each with a stated purity of 98%, were purchased from Sigma-Aldrich (St. Louis, MO, USA). HPLC-grade acetonitrile (ACN), methanol (MeOH), and n-hexane were obtained from J.T. Baker (Radnor, PA, USA). C18 sorbent and mixed-mode cation-exchange (MCX) cartridges were purchased from Agela Technologies (Tianjin, China). Ammonium acetate and ammonium hydroxide were obtained from Sigma-Aldrich. LC-MS-grade water, ACN, and formic acid supplied by Fisher Chemical (Pittsburgh, PA, USA) were used for mobile-phase preparation. Sample extracts were filtered through 13 mm, 0.2 μm polytetrafluoroethylene (PTFE) membrane filters (Whatman, Little Chalfont, UK).

Individual stock standard solutions of DAP, MAD, PYR, and ROX were prepared separately in MeOH at 100 μg/mL. Appropriate volumes of the four individual stock solutions were then combined and diluted to prepare a mixed standard solution containing 2 μg/mL of each analyte. Individual stock standard solutions of CAP and VCM were prepared separately in MeOH at 100 μg/mL. Working standard solutions at the required concentrations were prepared by serial dilution of the corresponding mixed or individual stock standard solutions with 50% MeOH in water (MeOH/water, 1:1, *v*/*v*). Individual stock solutions were stored in amber tubes at −20 °C, protected from light, and used within six months of preparation; the mixed standard solution was used within one month. Working solutions were prepared fresh before analysis and used on the day of preparation.

### 2.2. Sample Collection and Storage

Muscle samples were collected according to a sampling plan in 2024 and 2025 after the animals had been slaughtered as part of routine slaughterhouse operations at slaughterhouses located in nine administrative regions across Korea. No animals were slaughtered specifically for the purpose of this study. The number of samples allocated to each region was determined on the basis of regional slaughter throughput, and one muscle sample was collected from each animal. Samples were transported to the laboratory under refrigerated conditions.

**Figure 1:**
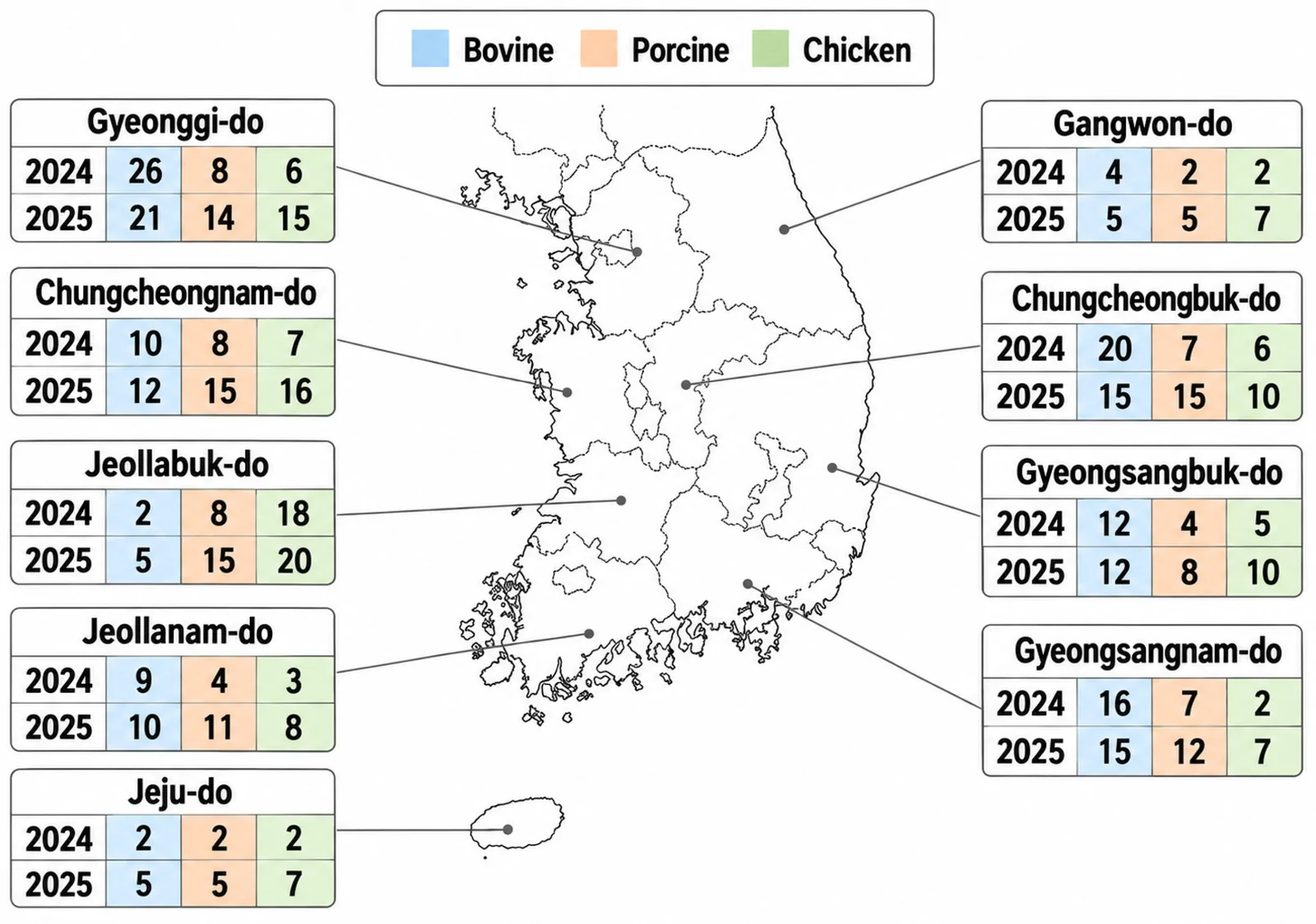
Sampling sites and numbers of livestock muscle samples collected in Korea during 2024–2025.

In 2024, 101 bovine, 50 porcine, and 51 chicken muscle samples (202 samples in total) were collected and analyzed for DAP, MAD, PYR, and ROX. In 2025, 100 muscle samples from each species (300 samples in total) were collected and analyzed for CAP and VCM. Thus, a total of 502 unique muscle samples were included across the two survey years. Upon arrival at the laboratory, the samples were homogenized, divided into 2 g aliquots, sealed, protected from light, and stored at −20 °C until analysis. The sampling regions and the numbers of samples collected for each species are presented in Figure 1.

### 2.3. Sample Preparation

#### 2.3.1. DAP, MAD, PYR, ROX, and CAP

Sample preparation for DAP, MAD, PYR, ROX, and CAP was performed according to the simultaneous multi-residue method for veterinary drugs in livestock and fishery products specified in Section 8.3.1 of the Korean Food Code [28].

A 2 g portion of homogenized muscle sample was weighed into a 50 mL centrifuge tube. Subsequently, 10 mL of water/ACN (1:4, *v*/*v*) was added, and the mixture was shaken for 5 min. After centrifugation at 4800× *g* for 10 min at 4 °C, the entire supernatant was transferred to a new 50 mL centrifuge tube.

C18 sorbent (500 mg) and 10 mL of ACN-saturated n-hexane were added to the supernatant. The mixture was shaken for 1 min and centrifuged at 4800× *g* for 5 min at 4 °C. A 5 mL portion of the lower phase was transferred to a new centrifuge tube and evaporated to dryness under a stream of nitrogen at 40 °C. The residue was reconstituted in 1 mL of water/MeOH (1:1, *v*/*v*), filtered through a 0.2 μm PTFE membrane filter, and transferred to a polypropylene vial for LC-MS/MS analysis.

#### 2.3.2. VCM

Sample preparation for VCM was performed according to the individual vancomycin method specified in Section 8.3.14 of the Korean Food Code [28].

A 2 g portion of homogenized muscle sample was weighed into a 50 mL centrifuge tube, and 10 mL of 15% ACN containing 50 mM ammonium acetate was added. The mixture was shaken for 10 min and centrifuged at 4000× *g* for 10 min at −4 °C. The supernatant was transferred to a new 50 mL centrifuge tube and designated extract A.

The remaining residue was re-extracted with 5 mL of the same extraction solvent, shaken for 10 min, and centrifuged under the same conditions. The second supernatant, designated extract B, was combined with extract A. Water-saturated n-hexane (6 mL) was added to the combined extract, and the mixture was shaken for 10 min and centrifuged at 4000× *g* for 10 min at −4 °C. The upper n-hexane layer was then discarded.

An MCX cartridge was conditioned sequentially with 3 mL of MeOH, 3 mL of water, and 3 mL of water containing 0.1% formic acid. The lower extract was loaded onto the conditioned cartridge. The cartridge was washed with 6 mL of water, and VCM was eluted with 6 mL of MeOH/ammonium hydroxide (97:3, *v*/*v*).

The eluate was evaporated to dryness under a stream of nitrogen at 40 °C. The residue was reconstituted in 1 mL of 10% ACN containing 0.1% formic acid, filtered through a 0.2 μm PTFE membrane filter, and transferred to a polypropylene vial for LC-MS/MS analysis.

### 2.4. LC-MS/MS Analysis

#### 2.4.1. DAP, MAD, PYR, ROX, and CAP

Chromatographic separation of DAP, MAD, PYR, ROX, and CAP was performed using an ExionLC system (AB SCIEX, Framingham, MA, USA). A Waters XBridge C18 column (2.1 mm × 150 mm, 3.5 μm) was used, and the column temperature was maintained at 40 °C. Mobile phases A and B consisted of water and ACN, respectively, each containing 0.1% formic acid. The flow rate was 0.3 mL/min, and the injection volume was 5 μL. The gradient program was defined using the following time-composition points: 0.0 min, 5% B; 0.5 min, 5% B; 5.5 min, 60% B; 6.0 min, 100% B; 10.0 min, 100% B; 10.2 min, 5% B; and 12.0 min, 5% B.

Mass spectrometric analysis was conducted using a QTRAP 5500 mass spectrometer equipped with an electrospray ionization source (AB SCIEX, Framingham, MA, USA). DAP, MAD, and PYR were analyzed in positive-ion mode, whereas ROX and CAP were analyzed in negative-ion mode. The ion-spray voltage was set to 5.5 kV in positive-ion mode and −4.5 kV in negative-ion mode. The source temperature was maintained at 600 °C. The nebulizing gas (GS1) and heating gas (GS2) pressures were set to 50 and 55 psi, respectively. Three multiple-reaction monitoring (MRM) transitions were optimized for each analyte. The precursor ions, product ions, collision energies, and retention times are presented in Table 2. Data acquisition and processing were performed using Analyst^®^ version 1.7.1 and MultiQuant™ version 3.0.3 (AB SCIEX, Framingham, MA, USA).

#### 2.4.2. VCM

Chromatographic separation of VCM was performed using a Nexera X2 system (Shimadzu, Kyoto, Japan) equipped with a Shim-pack GISS C18 column (2.1 mm × 150 mm, 3 μm). The column temperature was maintained at 40 °C, and the flow rate was set to 0.3 mL/min. Mobile phases A and B consisted of water and ACN, respectively, each containing 0.1% formic acid. The gradient program was defined using the following time-composition points: 0.0 min, 2% B; 0.5 min, 2% B; 8.0 min, 30% B; 8.5 min, 98% B; 10.0 min, 98% B; 10.1 min, 2% B; and 13.0 min, 2% B. The injection volume was 10 μL.

Mass spectrometric analysis was performed in positive electrospray-ionization mode using an LCMS-8050 triple-quadrupole mass spectrometer (Shimadzu, Kyoto, Japan). The interface, desolvation line, and heat-block temperatures were set to 300, 250, and 400 °C, respectively. The nebulizing, heating, and drying gas were supplied at flow rates of 3, 10, and 10 L/min, respectively. The MRM transitions used for the quantification and confirmation of VCM are presented in Table 2. Instrument control, data acquisition, and data processing were performed using LabSolutions version 5.96 (Shimadzu, Kyoto, Japan).

#### 2.4.3. Identification and Quantification Criteria

Identification of all target analytes was based on agreement of retention time with the corresponding reference standard and the presence of three MRM transitions. Quantification was performed using species-specific matrix-matched calibration curves. According to Codex Guideline CXG 71-2009, the relative ion intensities of the qualifier transitions in the samples were required to agree with those of the corresponding reference standards within relative tolerances of ≤20%, ≤25%, and ≤30% when the reference relative ion intensity was >50%, 20–50%, and 10–20%, respectively; for relative ion intensities <10%, a tolerance of ≤50% was applied [30].

### 2.5. Analytical Performance Verification

To establish suitability for application to bovine, porcine, and chicken muscle matrices under the laboratory conditions used in this study, the analytical performance of the existing official methods specified in the Korean Food Code was verified. The performance characteristics assessed were selectivity, linearity, limit of detection (LOD), LOQ, recovery, repeatability (intra-day precision), inter-day precision, and between-analyst performance. The verification design and acceptance criteria were established with reference to the method-verification framework of the Korean Food Code and Codex Guideline CXG 71-2009 [30]. For the concentration ranges evaluated in this study, the Codex concentration-dependent criteria for mean recovery were 50–120%, 60–120%, 70–120%, and 70–110% at ≤1, 1–10, 10–100, and 100–1000 μg/kg, respectively. The corresponding maximum repeatability CVs were 35%, 30%, 20%, and 15%, respectively. The corresponding overall within-laboratory CV benchmarks were 36%, 32%, 22%, and 18%, respectively, and these values were used as reference criteria for the inter-day precision assessment [30]. Because the target analytes are prohibited substances for which Codex Maximum Residue Limits for Veterinary Drugs (MRLVDs) have not been established, these concentration-dependent criteria were used as performance benchmarks for the fitness-for-purpose verification rather than as MRL-specific regulatory acceptance limits.

Blank muscle matrices were prepared separately for each species by pooling muscle samples collected from multiple regions. Each candidate sample was first screened for the target analytes, and only samples in which none of the relevant analytes were detected at or above the corresponding LOD were pooled and thoroughly homogenized. The resulting pooled homogenate was used as the species-specific blank matrix throughout the verification experiments.

Selectivity was evaluated by analyzing blank muscle samples from each species and examining whether interfering peaks were present at the retention times of the target analytes in the quantifier and qualifier MRM channels.

Linearity was evaluated using species- specific matrix-matched calibration standards prepared by fortifying blank samples before the complete sample-preparation procedure. The lower limit of the validated calibration range was defined as the lowest calibration level at or above the established analyte- and species-specific LOQ. Calibration curves were constructed using linear regression, and linearity was evaluated using the coefficient of determination (R^2^).

The LOD and LOQ were determined by fortifying blank muscle samples from each species with the target analytes and subjecting them to the complete sample-preparation and LC-MS/MS procedures. The LOD was defined as the lowest concentration producing a signal-to-noise ratio (S/N) of at least 3. The LOQ was defined as the lowest concentration producing an S/N of at least 10.

Recovery and precision were evaluated at 2, 10, and 50 μg/kg for DAP and MAD; 0.1, 0.5, and 2.5 μg/kg for PYR; 10 and 50 μg/kg for ROX in bovine muscle; 5, 25, and 125 μg/kg for ROX in porcine and chicken muscle; 0.15, 0.4, and 2.0 μg/kg for CAP; and 1, 2, and 10 μg/kg for VCM.

Recovery and precision were evaluated using three independently fortified replicates at each concentration level on each of three separate days (*n* = 9 per concentration level). Mean recovery was calculated from the nine results obtained at each concentration level. Repeatability was assessed from triplicate results obtained within a day, whereas inter-day precision was assessed from the nine results obtained across the three days. Precision was expressed as relative standard deviation (RSD).

Analyst-to-analyst comparability was evaluated separately by two analysts. Each analyst prepared an independent matrix-matched calibration curve and analyzed six independently fortified samples at a single concentration. The comparison concentrations were 1 μg/kg for PYR and CAP, 5 μg/kg for VCM, and 20 μg/kg for DAP, MAD, and ROX. Differences in variance and mean recovery between analysts were evaluated using an F-test and an independent-samples t-test, respectively, with statistical significance set at *p* < 0.05. These comparisons were used only to assess analyst-to-analyst consistency and were not used to calculate the concentration-level recovery values reported for method performance.

To provide an external assessment of laboratory performance, the laboratory participated in a proficiency test for the determination of CAP in bovine muscle organized by Test Veritas S.r.l. (Padova, Italy) in 2025. Performance was evaluated using the z-score assigned by the proficiency-testing provider. A result was considered satisfactory when |z| ≤ 2.

### 2.6. Application to Nationwide Monitoring and Quality Control

Each analytical batch included a solvent blank, a matrix blank, species-specific matrix-matched calibration standards, and a fortified quality-control sample to verify the acceptability of the analytical procedure. For monitoring purposes, a sample was classified as a reportable positive result when the retention-time and MRM-transition identification criteria were satisfied and the calculated concentration was equal to or greater than the applicable analyte-and species-specific LOQ. Results below the LOQ were treated as “not detected” for regulatory reporting purposes in accordance with the Korean Food Code.

## 3. Results and Discussion

### 3.1. Analytical Performance of the Methods

In this study, the analytical performance of LC-MS/MS methods was verified for five prohibited target substances and one major metabolite of DAP in bovine, porcine, and chicken muscle matrices. The performance characteristics evaluated included selectivity, linearity, LOD, LOQ, recovery, repeatability, inter-day precision, and between-analyst performance. The results are summarized in Table 3, with detailed concentration-level recovery and precision data provided in Table S2. All analyte-matrix combinations evaluated in this study met the predefined performance acceptance criteria.

**Figure 2:**
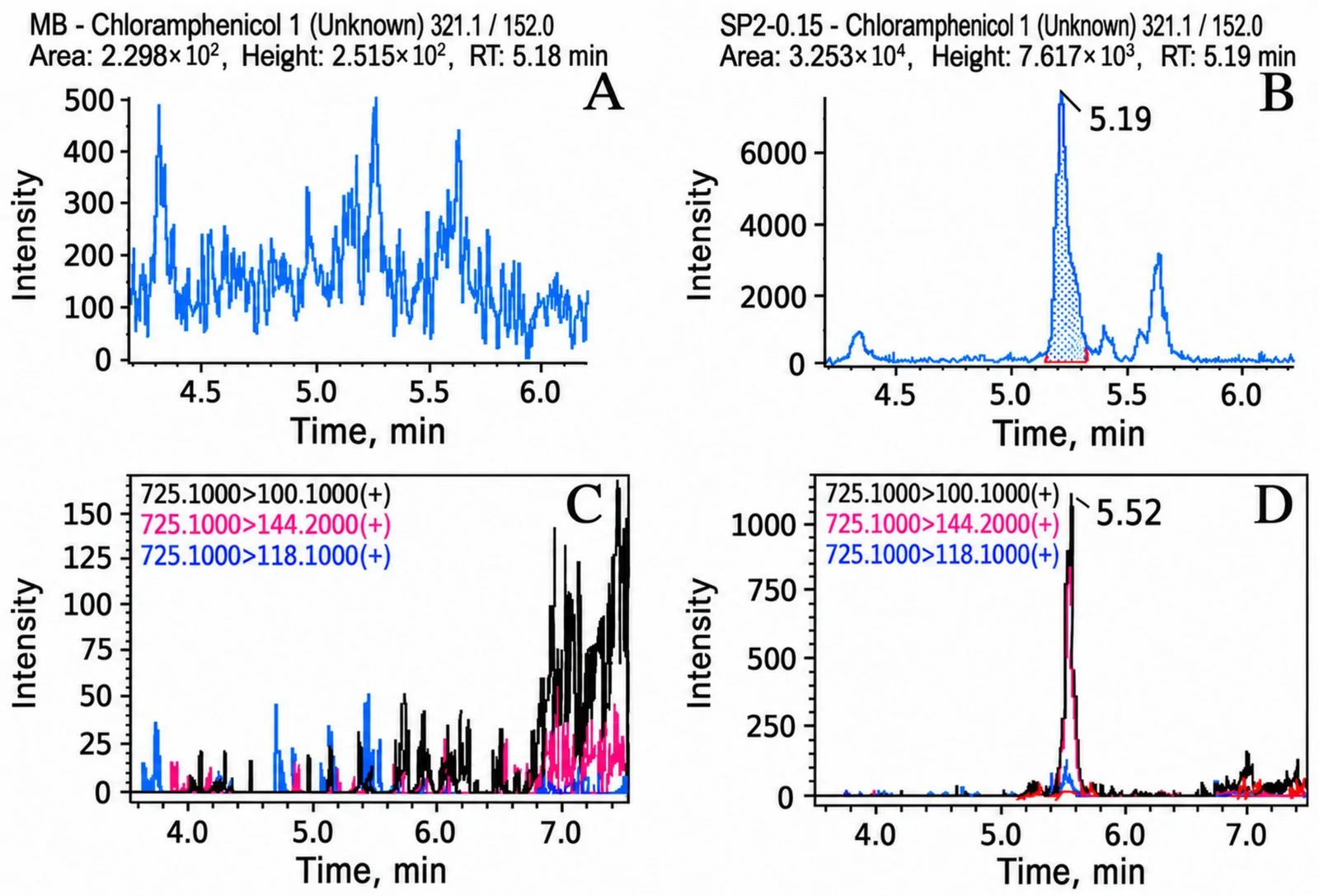
Representative MRM chromatograms of chloramphenicol (CAP) and vancomycin (VCM) in porcine muscle: (**A**) blank matrix for CAP; (**B**) CAP-fortified matrix at LOQ; (**C**) blank matrix for VCM; (**D**) VCM-fortified matrix at LOQ.

No interfering peaks were observed at the retention times of the target analytes in blank muscle samples from any of the three species. In samples fortified at the respective LOQs, the analyte peaks were clearly distinguishable from the background. Representative MRM chromatograms of the target analytes are presented in Figure 2, Figure S1 and Figure S2.

The LOQ for DAP was 1.0 μg/kg in all three species, whereas that for MAD ranged from 1.0 to 2.0 μg/kg, depending on the species. The LOQ for PYR was 0.1 μg/kg in bovine and chicken muscle and 0.05 μg/kg in porcine muscle, representing the lowest LOQ among the evaluated analytes. The LOQ for ROX was 3.5 μg/kg in bovine muscle and 4.0 μg/kg in porcine and chicken muscle. VCM had an LOQ of 1.0 μg/kg in all three species.

The LOQ for CAP was 0.15 μg/kg in bovine, porcine, and chicken muscle. This concentration is approximately one-third of the LOQ of 0.5 μg/kg specified for the Korean Food Code method and is equivalent to the EU RPA for CAP. Thus, the method provided quantitative sensitivity at the EU RPA. However, this result should be interpreted only as demonstrating quantitative capability at the RPA and does not indicate that all requirements for decision limit (CCα), identification, and confirmatory analysis established under Commission Implementing Regulation (EU) 2021/808 were evaluated or fulfilled [31].

Linearity was evaluated by linear regression using species-specific matrix-matched calibration curves. The R^2^ values were ≥0.997 for all analyte–matrix combinations. Species-specific matrix-matched calibration was used to minimize matrix-related bias in quantification; however, matrix effects were not independently quantified, and stable isotope-labeled internal standards were not employed.

Mean recoveries ranged from 92% to 106% across all analytes, matrices, and fortification levels and within the applicable concentration-dependent recovery ranges specified in CXG 71-2009 [30]. Repeatability and inter-day RSDs were ≤10.12% across all analyte-matrix-concentration combinations and were below the corresponding concentration-dependent precision benchmarks defined in Section 2.5. The highest inter-day RSD was 10.12% for ROX in porcine muscle at 5 μg/kg, the lowest fortification level evaluated for this analyte-matrix combination (Table S2). This value remained within the applicable precision criterion. In the between-analyst assessment, mean recoveries ranged from 95% to 106%, and neither the variances nor the mean recoveries differed significantly between analysts (all F-test and *t*-test *p*-values > 0.05; Table S2).

Overall, the findings demonstrate that the methods provided fit-for-purpose analytical performance for application to nationwide monitoring of the investigated target analytes in bovine, porcine, and chicken muscle matrices.

### 3.2. Chloramphenicol Proficiency Testing

In the proficiency test for CAP in bovine muscle, the laboratory obtained a z-score of −0.30, which was within the satisfactory range (|z| ≤ 2). This result demonstrated satisfactory agreement with the assigned value and acceptable laboratory performance in an external proficiency-testing exercise involving a matrix directly relevant to the present study. The proficiency-test result is presented in Figure S3.

A z-score of 0.85 was also obtained for raw milk and was within the satisfactory range. However, because raw milk was not one of the matrices investigated in this study, this result was regarded only as supplementary evidence of laboratory performance and was not used to support the performance claims for muscle matrices.

The assigned value for the bovine muscle proficiency-test material was 1.46 μg/kg, whereas the LOQ established in this study was 0.15 μg/kg. The performance demonstrated at the proficiency-test concentration should therefore be distinguished from method performance at the LOQ. Recovery and precision at 0.15 μg/kg were assessed separately through within-laboratory verification experiments using fortified muscle samples, as shown in Table S2.

Moreover, the proficiency test was conducted only for CAP. Consequently, the satisfactory proficiency-test result cannot be extrapolated to demonstrate the external analytical performance of the methods for DAP, MAD, PYR, ROX, or VCM.

### 3.3. Application to Nationwide Meat Monitoring

Under the Korean Food Code, substances designated as prohibited must not be detected in food, and results below the established LOQ are treated as “not detected” for regulatory reporting purposes [9]. Accordingly, the analyte- and species-specific LOQ was used in the present monitoring study as the regulatory reporting threshold for classifying monitoring results. This regulatory use of the LOQ should be distinguished from the analytical LOD, which represents the lower level at which the presence of an analyte can be detected without necessarily allowing reliable quantification. A sample was classified as a reportable positive result only when the identification criteria were satisfied and the calculated concentration was equal to or greater than the applicable analyte- and species-specific LOQ. No sample met this regulatory reporting criterion for any target analyte included in the respective annual surveys (Table S3). Thus, no quantifiable and analytically confirmed residues of the target analytes were observed in the production-stage muscle samples examined. The findings are limited to bovine, porcine, and chicken muscle samples collected in 2024 and 2025 and should not be generalized to other animal-derived foods or other time periods.

### 3.4. Comparison with Previously Reported Analytical Methods and Monitoring Data

Comparison with previous studies is useful for evaluating the analytical sensitivity and practical applicability of the methods used in the present study. However, the reported performance parameters differed among studies. Some studies reported LODs and LOQs, whereas others reported decision limits (CCα) or detection capabilities (CCβ).

Because LOD/LOQ and CCα/CCβ differ in their definitions, purposes, and statistical bases, direct one-to-one conversion or absolute ranking of methods based on these parameters is inappropriate. Tables S4–S8 therefore present the different performance parameters separately. The following discussion considers not only the reported numerical values but also the target analytes, matrices, sample-preparation procedures, analytical objectives, and applicability of each method to routine monitoring.

#### 3.4.1. DAP and MAD

DAP has a history of use in certain areas of animal production but is currently not authorized for use in food-producing animals in several regulatory jurisdictions. In the present study, the LOQ for DAP was 1.0 μg/kg in all three species, whereas the LOQ for MAD ranged from 1.0 to 2.0 μg/kg.

Dedicated methods developed specifically for DAP have reported lower CCα or CCβ values [32,33]. However, studies applying multi-residue methods to meat have also reported quantification levels similar to or higher than those obtained in the present study [34,35,36]. Direct comparison remains limited because the reported performance parameters and analytical objectives differ among studies.

The present method was not designed for ultra-trace, single-analyte analysis. Rather, its practical value lies in the simultaneous determination of the parent compound and its major metabolite using an existing official method and its application to nationwide monitoring of production-stage meat samples. Comparative data for DAP and MAD are presented in Table S4 [32,33,34,35,36,37,38,39,40].

#### 3.4.2. PYR

The LOQ for PYR was 0.1 μg/kg in bovine and chicken muscle and 0.05 μg/kg in porcine muscle. These LOQs were generally comparable to or lower than those reported for previous multi-residue LC-MS/MS methods [41,42]. The method also enabled quantification at lower concentrations than a previously reported fluorescence-detection method [43].

The sensitivity achieved in the present study was sufficient for monitoring PYR in production-stage meat according to the predefined performance criteria. Nevertheless, differences among studies in matrices, sample amounts, sample preparation procedures, concentration factors, and reported performance parameters limit direct comparison and preclude ranking the methods solely on the basis of their reported sensitivity. Comparative results are presented in Table S5 [41,42,43,44,45].

#### 3.4.3. ROX

ROX is an organoarsenical compound that was formerly used in poultry production for growth promotion and the control of coccidiosis. Food safety concerns related to ROX involve not only the parent compound but also other organoarsenic compounds and inorganic arsenic species that may arise through metabolic transformation in animals or transformation in the environment [18,20,22,46].

Because inorganic arsenic is carcinogenic to humans [47], parent-ROX residue analysis should be clearly distinguished from comprehensive arsenic-speciation analysis. Measurement of the parent compound alone does not provide information on the occurrence or concentrations of other ROX-related arsenic species.

In this study, the LOQ for ROX was 3.5 μg/kg in bovine muscle and 4.0 μg/kg in porcine and chicken muscle. Dedicated arsenic-speciation methods based on HPLC-ICP-MS or other LC-based techniques have reported lower LODs or LOQs for ROX or related arsenic species in chicken muscle [20,46,48]. Such methods, however, are specifically designed for the separation and sensitive determination of individual arsenic species.

In contrast, the present study used a Korean Food Code-based multi-residue LC-MS/MS procedure that enabled ROX to be analyzed together with other target compounds. Direct comparison of analytical sensitivity between these different method types is therefore limited. Nevertheless, the method met the predefined performance criteria for its intended application in routine residue monitoring. Comparative data are provided in Table S6 [20,46,48,49,50].

Accordingly, the absence of reportable positive results for ROX in this study indicates only that the parent compound did not meet the regulatory reporting criterion in the examined muscle samples. It does not exclude the possible presence of other organoarsenic compounds, ROX transformation products, or inorganic arsenic species.

#### 3.4.4. CAP

CAP is a well-recognized substance that is not authorized for use in food-producing animals because of its association with serious adverse effects in humans, including aplastic anemia [2,23,51]. Previous studies have reported analytical performance for CAP using different performance parameters, including LOD, LOQ, CCα, and CCβ, depending on the matrix and analytical procedure [52,53,54,55,56,57,58,59,60,61,62] (Table S7).

In the present study, an LOQ of 0.15 μg/kg was achieved in bovine, porcine, and chicken muscle, indicating that the official Korean Food Code method provided quantitative sensitivity at the EU RPA for CAP under the matrices and laboratory conditions evaluated. However, because not all performance and identification requirements specified in Commission Implementing Regulation (EU) 2021/808 were independently evaluated, these results should not be interpreted as demonstrating full compliance with that Regulation.

EU residue-monitoring reports covering 2020–2023 continued to document non-compliant findings involving CAP [63,64,65,66] (Table S9). The continued occurrence of such findings underscores the practical relevance of achieving quantitative capability at the EU RPA for regulatory surveillance of CAP residues in animal-derived foods.

#### 3.4.5. VCM

VCM is an important glycopeptide antimicrobial used in human medicine for the treatment of severe infections caused by Gram-positive bacteria [24,25,26]. In the present study, the LOQ for VCM was 1.0 μg/kg in bovine, porcine, and chicken muscle, which was broadly comparable to the analytical sensitivities reported in previous LC-MS/MS studies of VCM in tissues from food-producing animals and other food matrices [67,68,69,70,71,72] (Table S8). The achieved sensitivity met predefined performance criteria for the intended monitoring of VCM residues in muscle. Owing to its relatively high polarity, high molecular mass, and other physicochemical characteristics distinct from those of the other target analytes, VCM was analyzed using a separate extraction procedure, MCX solid-phase extraction cleanup, and distinct LC-MS/MS conditions. This highlights the practical challenges of incorporating prohibited substances with widely differing physicochemical properties into a single multi-residue analytical procedure.

#### 3.4.6. Previous Monitoring Studies in Korea

Various monitoring studies have investigated veterinary drug residues in livestock and fishery products in Korea. However, nationwide monitoring data focused specifically on substances prohibited for use in food-producing animals remain relatively limited. In previous Korean multi-residue monitoring studies, all residues reported as detected corresponded to veterinary drugs authorized for use in Korea; no detected residue was identified as a substance designated as prohibited under the Korean Food Code [73,74]. Similarly, no sample met the regulatory reporting criterion for DAP, MAD, PYR, ROX, CAP, or VCM in the present study. However, differences in sampling design, target analytes, matrices, analytical sensitivity, and survey periods should be considered when comparing these findings with previous studies.

The present study contributes additional domestic residue-monitoring data by evaluating the species-specific analytical performance of official Korean Food Code methods and subsequently applying them to 502 production-stage muscle samples. In particular, it provides baseline occurrence data for selected prohibited substances for which routine NRP monitoring data are currently limited.

### 3.5. Significance and Limitations of the Study

The principal significance of this study lies in its integration of laboratory-based analytical performance evaluation with practical application to nationwide production-stage samples. Although the study did not develop a new analytical principle or sample-preparation procedure, it evaluated existing official methods in bovine, porcine, and chicken muscle matrices and applied them to selected target substances for which routine monitoring data are limited. The resulting data are therefore relevant to the practical implementation of food safety surveillance for these target analytes. The LOQ of 0.15 μg/kg achieved for CAP in all three muscle matrices demonstrates quantitative capability at the EU RPA under the conditions evaluated in this study. In addition, the satisfactory result obtained in an external proficiency test using bovine muscle provides independent evidence of laboratory performance for CAP at the concentration represented by the test material. However, the proficiency-test result should not be interpreted as direct evidence of method performance at the EU RPA.

Several limitations should be considered. First, the analytical scope was restricted to five prohibited target substances and one major DAP metabolite, and the matrices were limited to bovine, porcine, and chicken muscle. The results therefore do not represent all prohibited substances or all livestock-derived foods. Second, analytical sensitivity in the present study was characterized using LOD and LOQ, whereas some previous studies reported CCα and CCβ. Because these parameters differ in their definitions, purposes, and statistical foundations, their numerical values are not directly comparable. Third, only the parent ROX was analyzed; therefore, the findings cannot be extrapolated to ROX-related transformation products, other organoarsenic compounds, or inorganic arsenic species. Fourth, species-specific matrix-matched calibration curves were used to minimize matrix-related bias; however, the extent of matrix-induced ion suppression or enhancement was not independently quantified, and stable isotope-labeled internal standards were not used. Therefore, analyte-specific matrix effects could not be fully characterized or corrected. Fifth, the monitoring design differed between the two survey years. DAP, MAD, PYR, and ROX were analyzed in 202 samples collected in 2024, whereas CAP and VCM were analyzed in 300 samples collected in 2025. Consequently, all six analytes were not evaluated in the same set of samples, and the unequal sample sizes and different analyte panels preclude direct comparison of residue occurrence among all target analytes or assessment of temporal changes between the two years. The monitoring findings should therefore be interpreted as analyte- and year-specific surveillance results rather than as an integrated comparison across the entire 2024–2025 dataset. This design reflects the annual targeted surveillance framework under which different analytes were selected for evaluation in each survey year.

Accordingly, the present study is best regarded as a matrix- and laboratory-specific fitness-for-purpose verification of existing official methods rather than a full validation of newly developed analytical methods. Despite these limitations, the study provides baseline analytical and occurrence data for selected prohibited substances in production-stage meat in Korea.

## 4. Conclusions

In this study, the analytical performance of official LC-MS/MS methods specified in the Korean Food Code for PYR, DAP, MAD, ROX, CAP, and VCM was verified in bovine, porcine, and chicken muscle matrices, followed by application to nationwide production-stage samples. All analyte–matrix combinations met the predefined acceptance criteria for selectivity, linearity, sensitivity, recovery, repeatability, and inter-day precision under the conditions evaluated, and the between-analyst comparisons showed no significant differences in variance or mean recovery. For CAP, an LOQ of 0.15 μg/kg was achieved in all three muscle matrices, demonstrating quantitative capability at the EU RPA. A satisfactory result was also obtained in an external proficiency test using bovine muscle.

No sample met the regulatory reporting criterion for any target analyte included in the respective annual survey. A total of 202 samples were analyzed for DAP, MAD, PYR, and ROX in 2024, whereas 300 samples were analyzed for CAP and VCM in 2025. Because the analyte panels and sample sets differed between the two survey years, the monitoring results should be interpreted on an analyte- and year-specific basis and not as a direct comparison of occurrence across analytes or between years. More broadly, these findings are limited to the sampling periods, species, matrices, and analytical scope of this study and should not be interpreted as demonstrating the complete absence of residues in other livestock-derived foods or time periods. Because only parent ROX was analyzed, the findings do not provide information on other ROX-related arsenic species.

Overall, the study demonstrates the fitness for purpose of existing Korean Food Code methods for the selected analytes under the evaluated laboratory and matrix conditions and provides baseline analytical and occurrence data to support residue surveillance in production-stage meat. Future work could extend the range of target substances and matrices and evaluate additional performance characteristics where required for specific regulatory applications.

## Figures and Tables

**Table 1:** Target prohibited veterinary drugs and related analytes included in this study.

No.	Compound	Korea Regulatory Status	NRP Routine Testing	Korean Food Code Section	Main Regulatory/Toxicological Concern
**1**	Pyrimethamine	Prohibited	No	8.3.1	Folate-antagonist hematotoxicity, including bone-marrow suppression; developmental toxicity
**2**	Dapsone	Prohibited	No	8.3.1	Methemoglobinemia, hemolytic anemia, and serious blood dyscrasias
**3**	Roxarsone	Prohibited	No	8.3.1	Potential conversion to inorganic arsenic and associated carcinogenicity concerns
**4**	Chloramphenicol	Prohibited	Yes	8.3.1	Serious hematotoxicity including aplastic anemia; prohibited in food-producing animals
**5**	Vancomycin	Prohibited	No	8.3.14	Human-only glycopeptide in the 2024 WHO MIA List; preservation of therapeutic effectiveness in human medicine

**Table 2:** Optimized multiple reaction monitoring parameters for target analytes.

Analyte	Ionization Mode	Precursor Ion (*m*/*z*)	Product Ion (*m*/*z*)	CE	RT (min)
DAP	positive	249	92 *	33	4.67
			156	25	
			108	25	
MAD	positive	291	92 *	35	4.84
			155.9	23	
			108.1	31	
PYR	positive	249.1	233.1 *	40	4.82
			177	40	
			198.1	55	
ROX	negative	262	244	20	3.12
			123.1 *	35	
			153.1	30	
CAP	negative	321.1	152 *	24	5.21
			257	15	
			194	17	
VCM ^†^	positive	725.1	100.1 *	40	5.50
			144.2	20	
			118.1	20	

Quantifier product ions are marked with an asterisk (*); the remaining product ions were used as qualifier ions. DAP, dapsone; MAD, monoacetyldapsone; PYR, pyrimethamine; ROX, roxarsone; CAP, chloramphenicol; VCM, vancomycin; CE, collision energy; RT, retention time. ^†^ VCM was analyzed using a different LC-MS/MS platform from that used for the other analytes.

**Table 3:** Summary of analytical performance of the official methods in bovine, porcine, and chicken muscle.

Analyte	Matrix	LOD/LOQ (µg/kg)	Calibration Range (µg/kg)	R^2^	Mean Recovery Range (%)	Repeatability RSD Range (%)	Inter-Day RSD Range (%)
DAP	Bovine	0.5/1.0	1–100	0.9994	97–101	2.21–5.85	7.28–9.91
	Porcine	0.2/1.0	1–100	0.9995	96	1.76–3.35	3.19–4.30
	Chicken	0.2/1.0	1–100	0.9995	100–103	2.19–3.43	2.31–5.62
MAD	Bovine	1.0/2.0	2–100	0.9992	97–100	0.42–0.90	3.10–5.22
	Porcine	0.5/1.0	1–100	0.9999	95–99	1.71–3.19	3.59–6.22
	Chicken	0.5/2.0	2–100	0.9990	98–104	2.32–7.78	3.49–8.02
PYR	Bovine	0.025/0.1	0.1–5	0.9995	95–100	3.03–6.00	4.43–5.62
	Porcine	0.02/0.05	0.05–5	0.9998	96–101	1.89–5.91	4.97–6.45
	Chicken	0.02/0.1	0.1–5	0.9986	94–101	1.47–6.19	3.41–9.34
ROX	Bovine	2.0/3.5	3.5–100	0.9994	98–99	2.11–4.83	3.75–5.81
	Porcine	1.0/4.0	4–250	0.9978	94–101	2.25–8.12	1.37–10.12
	Chicken	1.0/4.0	4–250	0.9997	100–106	2.08–7.45	4.13–9.37
CAP	Bovine	0.05/0.15	0.15–5	0.9994	99–100	2.87–4.23	4.70–6.28
	Porcine	0.05/0.15	0.15–5	0.9999	92–99	0.99–4.17	3.78–8.30
	Chicken	0.05/0.15	0.15–5	0.9992	98–100	1.44–4.89	1.90–4.01
VCM	Bovine	0.3/1.0	1–20	0.9990	100–106	1.57–4.47	3.13–4.52
	Porcine	0.3/1.0	1–20	0.9986	99–105	1.86–3.84	3.75–4.28
	Chicken	0.3/1.0	1–20	0.9990	98–103	2.06–6.71	3.87–6.01

DAP, dapsone; MAD, monoacetyldapsone; PYR, pyrimethamine; ROX, roxarsone; CAP, chloramphenicol; VCM, vancomycin; LOD, limit of detection; LOQ, limit of quantification; R^2^, coefficient of determination. Recovery ranges represent the minimum-to-maximum mean recoveries calculated from nine determinations at each fortification level over three days (*n* = 9). Repeatability RSDs were based on triplicate within-day measurements (*n* = 3), and inter-day RSDs were based on nine measurements across three days (*n* = 9). Detailed concentration-level recovery and precision results, together with the separate analyst-to-analyst comparison, are provided in Table S2.

## Data Availability

The aggregated data supporting the findings of this study are included in the article and Supplementary Materials. Individual sample-level monitoring data are not publicly available because they are subject to institutional and regulatory data-access restrictions. De-identified data may be available from the corresponding author upon reasonable request and subject to approval by the Animal and Plant Quarantine Agency.

## Supplementary Materials

The following supporting information can be downloaded at https://www.mdpi.com/article/10.3390/foods15203662/s1: Table S1, Substances designated in the Korean Food Code as substances that must not be detected in food; Table S2, Detailed concentration-level recovery and precision data for target analytes in bovine, porcine, and chicken muscle, including analyst-to-analyst comparison results; Figure S1, Representative MRM chromatograms of dapsone (DAP) and monoacetyldapsone (MAD) in porcine muscle: (A) blank matrix for DAP; (B) DAP-fortified matrix at the LOQ; (C) blank matrix for MAD; (D) MAD-fortified matrix at the LOQ; Figure S2, Representative MRM chromatograms of pyrimethamine (PYR) and roxarsone (ROX) in porcine muscle: (A) blank matrix for PYR; (B) PYR-fortified matrix at the LOQ; (C) blank matrix for ROX; (D) ROX-fortified matrix at the LOQ; Figure S3, Proficiency-testing results for chloramphenicol in (A) raw milk (z-score = 0.85; laboratory code T011) and (B) bovine muscle (z-score = −0.30; laboratory code T005); Table S3, Nationwide monitoring results for target prohibited veterinary drugs and related analytes in livestock muscle samples collected in Korea during 2024–2025; Table S4, Comparison with previously reported analytical methods for dapsone and monoacetyldapsone; Table S5, Comparison with previously reported analytical methods for pyrimethamine; Table S6, Comparison with previously reported analytical methods for roxarsone; Table S7, Comparison with previously reported analytical methods for chloramphenicol; Table S8, Comparison with previously reported analytical methods for vancomycin; Table S9, Non-compliant findings for chloramphenicol reported in EU residue-monitoring programs during 2020–2023.

## Abbreviations

The following abbreviations are used in this manuscript:

NRP	National Residue Program
PYR	Pyrimethamine
DAP	Dapsone
ROX	Roxarsone
CAP	Chloramphenicol
VCM	Vancomycin
MAD	Monoacetyldapsone
LOD	Limit of detection
LOQ	Limit of quantification
RPA	Reference point for action
ACN	Acetonitrile
MeOH	Methanol
MCX	Mixed-mode cation- exchange
PTFE	Polytetrafluoroethylene
MRM	Multiple-reaction monitoring
CE	Collision energy
RT	Retention time
S/N	Signal-to-noise ratio
RSD	Relative standard deviation
R^2^	Coefficient of determination
CCα	Decision limit
CCβ	Detection capability
