# Supplementary material for: Verification of Official LC-MS/MS Methods and Nationwide Monitoring of Five Prohibited Veterinary Drugs and a Major Metabolite in Livestock Muscle in Korea
Source: Foods. 2026 Oct 15;15(20):3662. doi: 10.3390/foods15203662 (PMC13703556; doi:10.3390/foods15203662)
Supplement: Supplementary file 1 [file foods-15-03662-s001.zip › foods-4575054-supplementary.pdf]

Table S1. Substances designated in the Korean Food Code as substances that must not be detected in food.

No.	Compound	MFDS notification	Action date	NRP routine monitoring	Korean Food Code section
1	Furazolidone	2007-68	2008-04-01	Yes	8.3.7
	Furaltadone	2007-68	2008-04-01	Yes	8.3.7
	Nitrofurazone	2007-68	2008-04-01	Yes	8.3.7
	Nitrofurantoin	2007-68	2008-04-01	Yes	8.3.7
	Nitrovin	2007-68	2008-04-01	No	8.3.6
2	Carbadox	2014-146	2014-09-11	Yes	8.3.1
3	Olaquindox	2014-146	2014-09-11	Yes	8.3.1
4	Chloramphenicol	2007-68	2008-04-01	Yes	8.3.1
5	Chlorpromazine	2007-68	2008-04-01	No	8.3.1
6	Clenbuterol	2007-68	2008-04-01	Yes	8.3.1
7	Colchicine	2007-68	2008-04-01	No	8.3.1
8	Dapsone	2014-146	2014-09-11	No	8.3.1
9	Diethylstilbestrol (DES)	2007-68	2008-04-01	Yes	8.3.10
10	Medroxyprogesterone acetate (MPA)	2007-68	2008-04-01	Yes	8.3.10
11	Thiouracil	2007-68	2008-04-01	No	8.3.26
12	Gentian violet, Crystal violet	2019-16	2020-04-01	No	8.3.12
13	Malachite green	2007-68	2008-04-01	No	8.3.12
14	Methylene blue	2019-16	2020-04-01	No	8.3.12
15	Dimetridazole	2007-68	2008-04-01	Yes	8.3.1
16	Ipronidazole	2016-154	2018-01-01	Yes	8.3.1
17	Metronidazole	2014-146	2014-09-11	Yes	8.3.1
18	Ronidazole	2014-146	2014-09-11	Yes	8.3.1
19	Norfloxacin	2019-16	2020-04-01	Yes	8.3.1
20	Ofloxacin	2019-16	2020-04-01	Yes	8.3.1
21	Pefloxacin	2019-16	2020-04-01	Yes	8.3.1
22	Pyrimethamine	2007-68	2008-04-01	No	8.3.1
23	Vancomycin	2007-68	2008-04-01	No	8.3.14
24	Roxarsone	2019-89	2020-10-14	No	8.3.1
25	Arsanilic acid	2019-89	2020-10-14	No	8.3.45
26	Salbutamol	2021-26	2022-04-01	No	8.3.23
27	Strychnine	2024-71	2024-11-15	No	8.3.74
28	Carbimazole /Thiamazole	2024-71	2024-11-15	No	8.3.75
29	Phenacetin	2025-56	2025-10-01	No	8.3.1
30	Phenylbutazone	2026-40	2026-05-19	Yes	8.3.28

Table S2. Detailed concentration-level recovery and precision data for target analytes in bovine, porcine, and chicken muscle, including analyst-to-analyst comparison results.

Analyte	Matrix	Fortification level (μg/kg)	Mean recovery (%, n=9)	Repeatability RSD (%, n=3)	Inter-day RSD (%, n=9)	F-test p-value	t-test p-value
DAP	Bovine	2	99	4.42	7.28	0.3972	0.7249
		10	97	5.85	7.63		
		50	101	2.21	9.91		
	Porcine	2	96	1.76	3.19	0.1050	0.5567
		10	96	3.35	3.83		
		50	96	1.88	4.30		
	Chicken	2	103	2.19	5.62	0.4064	0.4432
		10	100	3.08	4.38		
		50	103	3.43	2.31		
MAD	Bovine	2	100	0.86	5.22	0.3062	0.1469
		10	97	0.42	3.67		
		50	97	0.90	3.10		
	Porcine	2	95	3.19	6.22	0.4483	0.7217
		10	99	2.66	3.59		
		50	99	1.71	4.18		
	Chicken	2	98	7.78	8.02	0.2394	0.5383
		10	104	5.09	5.45		
		50	104	2.32	3.49		
PYR	Bovine	0.1	95	4.97	5.62	0.2338	0.1906
		0.5	100	6.00	5.09		
		2.5	98	3.03	4.43		
	Porcine	0.1	99	5.91	6.45	0.3971	0.1772
		0.5	101	5.62	4.97		
		2.5	96	1.89	6.04		
	Chicken	0.1	94	6.19	9.34	0.3076	0.7357
		0.5	101	4.28	4.97		
		2.5	98	1.47	3.41		
ROX	Bovine	10	98	4.83	5.81	0.4311	0.1106
		50	99	2.11	3.75		
	Porcine	5	99	8.12	10.12	0.3181	0.3484
		25	94	5.30	7.43		
		125	101	2.25	1.37		
	Chicken	5	100	7.45	9.37	0.2992	0.7234
		25	101	2.08	4.60		
		125	106	2.61	4.13		
CAP	Bovine	0.15	99	4.23	6.28	0.1885	0.7357
		0.4	100	3.63	4.70		
		2.0	100	2.87	5.56		
	Porcine	0.15	92	4.17	8.30	0.4344	0.3409
		0.4	98	1.44	5.16		
		2.0	99	0.99	3.78		
	Chicken	0.15	98	1.44	3.69	0.3238	0.0924
		0.4	98	1.79	1.90		
		2.0	100	4.89	4.01		
VCM	Bovine	1	106	4.47	3.67	0.2678	0.6647
		2	101	2.32	3.13		
		10	100	1.57	4.52		
	Porcine	1	105	3.84	3.83	0.3831	0.5709
		2	100	1.86	3.75		
		10	99	2.09	4.28		
	Chicken	1	103	3.22	6.01	0.2324	0.6435
		2	98	6.71	5.43		
		10	100	2.06	3.87		

DAP, dapsone; MAD, monoacetyldapsone; PYR, pyrimethamine; ROX, roxarsone; CAP, chloramphenicol; VCM, vancomycin; RSD, relative standard deviation.

Mean recovery was calculated from nine determinations at each fortification level over three days (n = 9). Repeatability RSD was based on triplicate within-day measurements (n = 3), and inter-day RSD was based on nine measurements across three days (n = 9).

F-test and t-test p-values were obtained from a separate analyst-to-analyst comparison in which two analysts each analyzed six independently fortified samples. Comparison concentrations were 20 μg/kg for DAP, MAD, and ROX; 1 μg/kg for PYR and CAP; and 5 μg/kg for VCM. $p < 0.05$ was considered statistically significant.

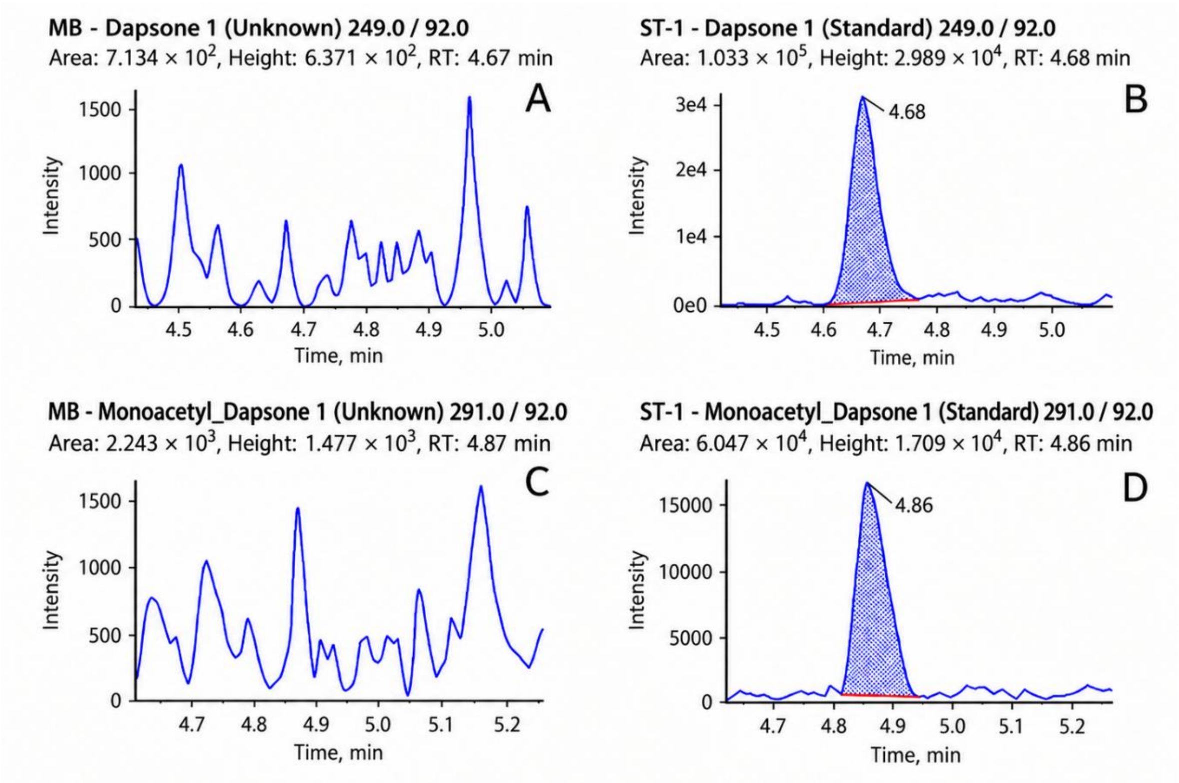

Figure S1. Representative MRM chromatograms of dapsone (DAP) and monoacetyldapsone (MAD) in porcine muscle: (A) blank matrix for DAP; (B) DAP-fortified matrix at the LOQ; (C) blank matrix for MAD; (D) MAD-fortified matrix at the LOQ.

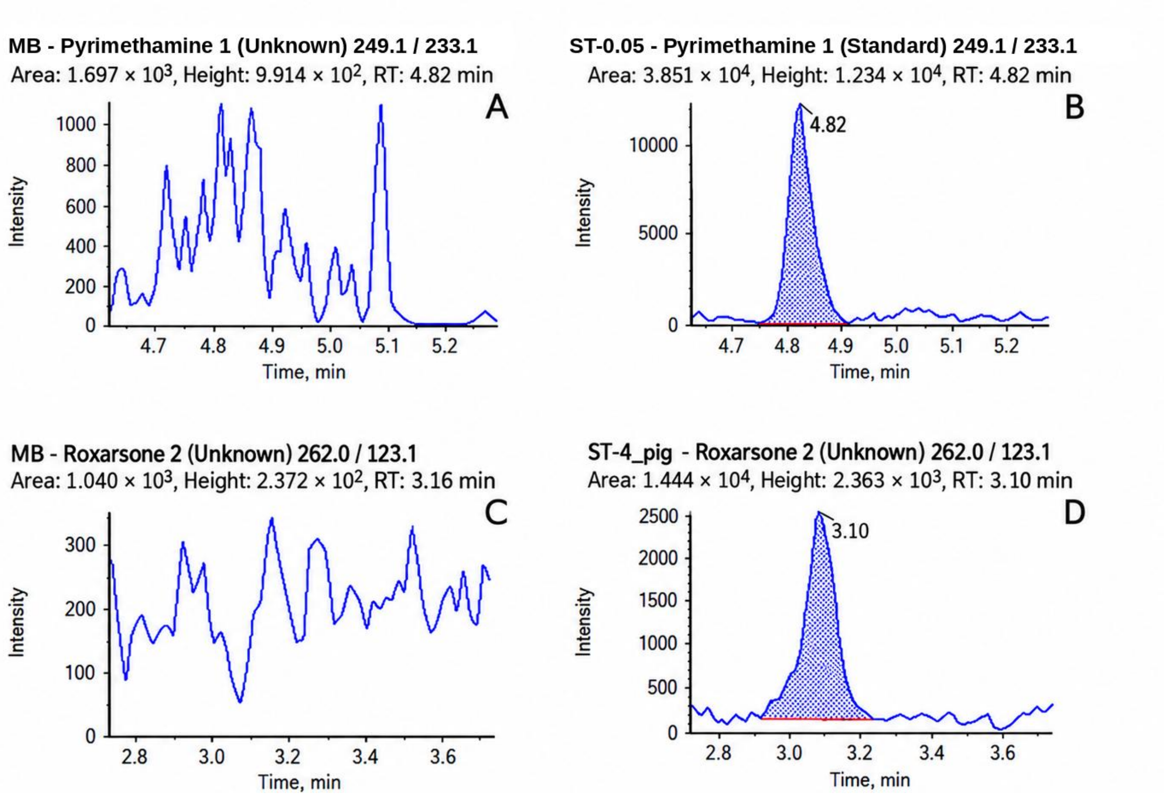

Figure S2. Representative MRM chromatograms of pyrimethamine (PYR) and roxarsone (ROX) in porcine muscle: (A) blank matrix for PYR; (B) PYR-fortified matrix at the LOQ; (C) blank matrix for ROX; (D) ROX-fortified matrix at the LOQ.

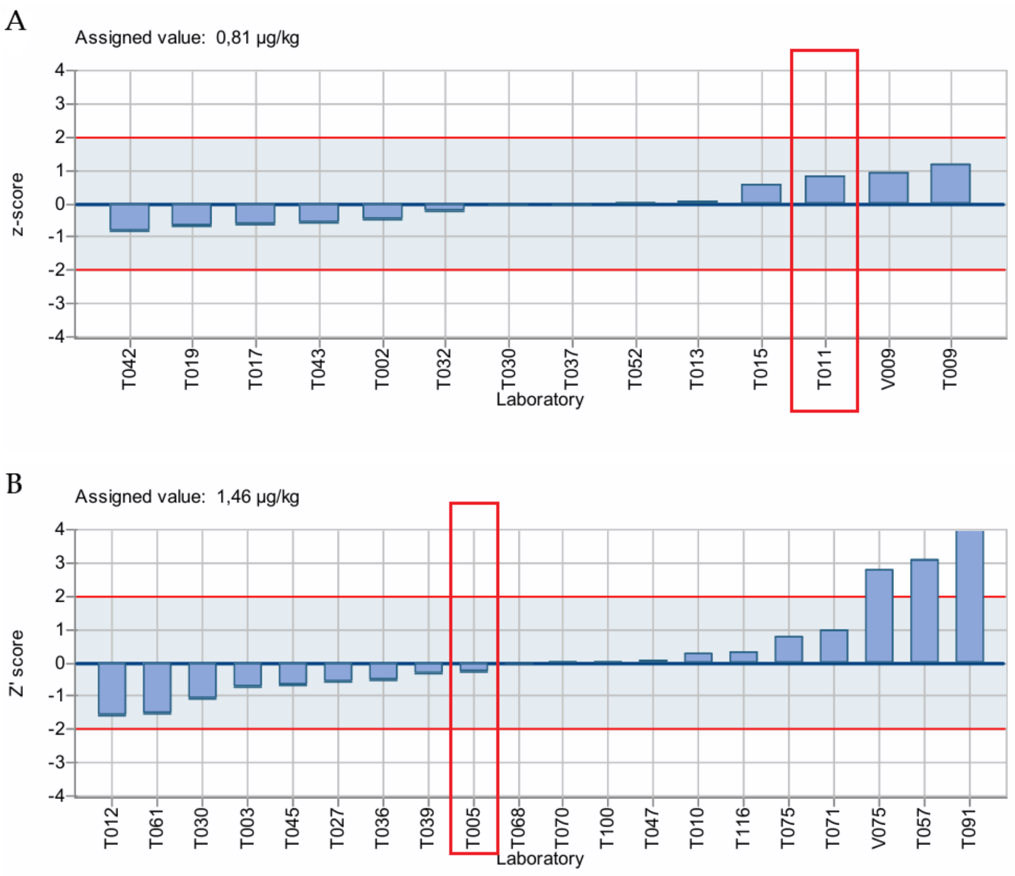

Figure S3. Proficiency-testing results for chloramphenicol in (A) raw milk (z-score = 0.85; laboratory code T011) and (B) bovine muscle (z-score = –0.30; laboratory code T005).

Table S3. Nationwide monitoring results for target prohibited veterinary drugs and related analytes in livestock muscle samples collected in Korea during 2024–2025

Region	2024 (DAP, MAD, PYR, and ROX)			2025 (CAP and VCM)		
	Bovine	Porcine	Chicken	Bovine	Porcine	Chicken
Gyeonggi-do	0/26	0/8	0/6	0/21	0/14	0/15
Gangwon-do	0/4	0/2	0/2	0/5	0/5	0/7
Chungcheongnam-do	0/10	0/8	0/7	0/12	0/15	0/16
Chungcheongbuk-do	0/20	0/7	0/6	0/15	0/15	0/10
Jeollabuk-do	0/2	0/8	0/18	0/5	0/15	0/20
Jeollanam-do	0/9	0/4	0/3	0/10	0/11	0/8
Gyeongsangbuk-do	0/12	0/4	0/5	0/12	0/8	0/10
Gyeongsangnam-do	0/16	0/7	0/2	0/15	0/12	0/7
Jeju-do	0/2	0/2	0/2	0/5	0/5	0/7
Total	0/101	0/50	0/51	0/100	0/100	0/100

Results are expressed as the number of samples meeting the regulatory reporting criterion/ number of samples analyzed.
For monitoring purposes, a sample was classified as a reportable positive result when the identification criteria were satisfied and the calculated concentration was equal to or greater than the applicable analyte- and species-specific LOQ; results below the LOQ were treated as “not detected” in accordance with the Korean Food Code [9].

Table S4. Comparison with previously reported analytical methods for dapsone and monoacetyldapsone

Matrix	Sample extraction and cleanup	Analytical technique	Calibration/linear range (μg/kg)	Recovery (%)	LOD/LOQ or CCα/ CCβ (μg/kg)	Reference
Muscle tissue, milk	Organic-solvent extraction followed by HLB cartridge	HPLC-MS/MS	0.005-10	Meat 105-117<br>Milk 101-108	CCα 0.0018<br>CCβ 0.0031	Kaklamanos et al. [32]
Meat, milk	Dichloromethane extraction at pH 10 and silica-gel SPE cleanup	LC-MS/MS	0.15-5.25	89.6-108	CCα 0.12<br>CCβ 0.16	Hadjigeorgiou et al. [33]
Bovine, porcine, ovine, poultry muscle<br>(21 analytes)	0.1 M EDTA + acetonitrile extraction, no SPE cleanup	UPLC-MS/MS	0-5	98.4	Bovine, porcine, ovine, poultry CCα/CCβ<br>(3.3/4.1; 3.4/4.1; 3.6/4.4; 3.4/4.2 respectively)	McDonald et al. [34]
Beef, pork, chicken, egg, milk (24 analytes)	ACN extraction with $MgSO_4$, sodium acetate + C_{18} cleanup	LC-MS/MS	1-100	79.1-117	Beef, pork, chicken, milk 1 (LOQ)<br>Egg 10 (LOQ)	Park & Kang [35]
Muscle, egg, milk, honey (15 analytes)	Acetone/dichloromethane extraction with SO_3H SPE cleanup	LC-MS/MS	Muscle, milk 0.5-1.5<br>Egg 2.5-7.5<br>Honey 1.25-3.75	93.2-103.7	CCα/CCβ:<br>Egg 2.8/3.1<br>Milk 0.5/0.6<br>Muscle 0.6/0.7<br>Honey 1.4/1.5	Varenina et al. [36]
Bovine milk<br>(16 analytes)	Acid hydrolysis + derivatization, then dispersive SPE + liquid-liquid extraction	LC-MS/MS	1.25-10	99-101	CCα 0.62<br>CCβ 1.23	Bongers et al. [37]
Chicken eggs<br>(244 analytes)	5% formic acid in acetonitrile extraction; EMR-Lipid d-SPE cleanup plus EMR polish	LC-MS/MS	0.1-100	61.9-66.6	LOD 0.2; LOQ 0.5;<br>CCα 8.5; CCβ 16.5	Zhang et al. [38]
Honey (11 analytes)	Acidic hydrolysis of honey to release sugar-bound residues, then Oasis HLB SPE	LC-MS/MS	0.5-100	70-74	LOD 0.4<br>CCα 0.9<br>CCβ 1.4	Economou et al. [39]
Milk powder, butter, fish tissue, eggs<br>(115 analytes)	H2O (with 0.1% FA and 0.1% EDTA(w/v)): ACN: MeOH (1:1:1, v/v) extraction and freezing followed defatting with hexane	LC-MS/MS	1-200	54.2-87.1	LOQ:<br>Butter 0.58<br>Egg 0.39<br>Fish tissue 0.73<br>Milk powder 0.38	Dasenaki & Thomaidis [40]
Beef, pork, chicken	Extraction ACN-H_2O (4:1, v/v), cleanup C18 and hexane saturated with ACN	LC-MS/MS	1-100 (DAP)<br>Porcine: 1-100 (MAD)<br>Bovine, Chicken: 2-100 (MAD)	DAP: 96-103<br>MAD: 95-104	LOD/LOQ:<br>Bovine 0.5/1 (DAP); 1/2 (MAD)<br>Porcine 0.2/1 (DAP); 0.5/1 (MAD)<br>Chicken 0.2/1 (DAP); 0.5/2 (MAD)	This study

Table S5. Comparison with previously reported analytical methods for pyrimethamine

Matrix	Sample extraction and cleanup	Analytical technique	Calibration/linear range (μg/kg)	Recovery (%)	LOD/LOQ or CCα/ CCβ (μg/kg)	Reference
Bovine, porcine, chicken muscle (132 analytes)	ACN: MeOH (95:5, v/v) extraction and defatting with hexane saturated with ACN	LC-ESI-MS/MS	3-30	Bovine 78.5<br>Porcine 78.5<br>Chicken 79.1	LOD 1.0<br>LOQ 3.0	Yamada et al. [41]
Chicken, swine, bovine, fish muscle; egg; milk; porcine liver/ kidney (21 analytes)	ACN/MeOH (95:5) with 1% FA extraction, MgSO4/NaOAc addition, ACN-saturated n-hexane purification	LC-MS/MS	0.5-25	Chicken muscle 87.8-96.5; Porcine muscle 94.2-106.5; Bovine muscle 90.1-104.0; Fish muscle 96.4-108.0; Egg 92.5-100.7; Milk 99.5-107.8; Liver 88.3-106.6; Kidney 42.9-98.1	LOQ 0.5(all 8 matrices)	Chang et al. [42]
Animal tissue and egg (swine muscle, chicken muscle, chicken liver, egg)	ACN extraction and cleanup with hexane and fluorescent derivatization with chloroacetaldehyde	LC-FDA	1-100	61.0-81.2	LOD milk 10	Horii et al. [43]
Cow milk (10 analytes)	20% TCA and McIlvain buffer (pH~4.0) followed by Oasis HLB	LC-ESI-MS	1-300	70.46-78.52	MDL 0.51<br>MQL 0.97	Koesukwiwat et al. [44]
Chicken processed foods (deep-fried chicken, non-fried chicken cutlet) and chicken muscle (37 analytes)	Ethyl acetate/ ACN + salts extraction followed by SLE cartridge (diatomaceous earth) clean-up	LC-MS/MS	0.5-40	84-95	LOQ 0.4(all 3 matrices)	Yoshikawa et al. [45]
Beef, pork, chicken	Extraction ACN-H_2O (4:1, v/v), cleanup C18 and hexane saturated with ACN	LC-MS/MS	Porcine: 0.05-5<br>Bovine, Chicken: 0.1-5	Bovine 95-100<br>Porcine 96-101<br>Chicken 94-101	LOD/LOQ:<br>Bovine 0.025/0.1<br>Porcine 0.02/0.05<br>Chicken 0.02/0.1	This study

Table S6. Comparison with previously reported analytical methods for roxarsone

Matrix	Sample extraction and cleanup	Analytical technique	Calibration/linear range (μg/kg)	Recovery (%)	LOD/LOQ or CCα/ CCβ (μg/kg)	Reference
Chicken meat	Malonic acid treatment with H_2O_2	HPLC-ICP-MS	-	-	2(LOD)	Nachman et al. [20]
Chicken meat	Ultrasonication plus papain	HPLC-ICP-MS<br>HPLC-ESI-MS	0.1-100	89	1.2/3.2(HPLC-ICP-MS)	Liu et al. [46]
Chicken liver	Pepsin-assisted enzyme treatment	HPLC-ICP/MS<br>HPLC-ESI-MS	0.2-200	88-92	0.4(LOD, ICP-MS))	Peng et al. [48]
Chicken tissues (liver, meat)	CM-α-CD-Fe3O4	UPLC-MS/MS	0.2-10.0	93.4-108.7	0.1(LOD)	Jia et al. [49]
Chicken muscle	Derivatization with toluene-3,4-dithiol	UPLC-UV	200-5000	80.39	100/200	Eom et al. [50]
Beef, pork, chicken	Extraction ACN-H_2O (4:1, v/v), cleanup C18 and hexane saturated with ACN	LC-MS/MS	Bovine 3.5-100<br>Porcine, Chicken 4-250	Bovine 98-99<br>Porcine 94-101<br>Chicken 100-106	Bovine 2.0/3.5<br>Porcine 1.0/4.0<br>Chicken 1.0/4.0	This study

Table S7. Comparison with previously reported analytical methods for chloramphenicol

Matrix	Sample extraction and cleanup	Analytical technique	Calibration/linear range (µg/kg)	Recovery (%)	LOD/LOQ or CCα/ CCβ (µg/kg)	Reference
Meat, egg, honey, milk	ACN extraction / purification by chloroform	LC-MS/MS	0.2-5	90-102	CCα 0.02<br>CCβ 0.04	Rønning et al. [52]
Chicken muscle (4 analytes)	Ethyl acetate: NH_4OH (98:2, v/v) extraction/ Hexane defatting + Oasis MCX cartridge clean-up	LC-MS/MS	0.3–20 µg/kg	101.7–103.5	CCα 0.07<br>CCβ 0.11<br>LOD 0.1<br>LOQ 0.3	Zhang et al. (2008) [53]
Chicken muscle	Ethyl acetate extraction/ no purification	UPLC-MS/MS	0.1-0.4	97.3–104.0	CCα 0.10<br>CCβ 0.11	Yibar et al. [54]
Pig muscle (7 analytes)	Diethyl ether extraction after β-glucuronidase/sulfatase digestion/ purification by immunoaffinity column (IAC-CZ)	LC-MS/MS	0.1-25	74.5–105.0	LOD 0.04<br>LOQ 0.10	Wang et al. [55]
Poultry, swine, bovine muscle (4 analytes)	Ethyl acetate: ammonium hydroxide (98:2) extraction/ Evaporation + hexane fat removal	LC-MS/MS		102–103 (bovine);<br>105 (swine);<br>98 (poultry)	CCα/ CCβ:<br>bovine: 0.06/0.11<br>swine: 0.04/0.07<br>poultry: 0.1/0.2	Barreto et al. [56]
Bovine muscle (41 analytes)	ACN + EDTA extraction/ n-Hexane defatting	UPLC-MS/MS	0.15-0.9	105	CCα 0.07<br>CCβ 0.10	Freitas et al. [57]
Multiple matrices including muscle	Hydrolysis then acetonitrile extraction with NaCl/ QuEChERS partition	HPLC–MS/MS	0.10-2.4	91.1-108.0	CCα/ CCβ:<br>Milk: 0.10/0.12<br>Muscle: 0.10/0.12<br>Egg: 0.10/0.14	Śniegocki et al. [58]
Beef, pork, chicken (4 analytes)	EDTA + NH_4OH: H_2O (2:98, v/v) + ACN extraction / PSA + $MgSO_4$ purification	LC–MS/MS	0.5-5	83.61–115.49 (beef);<br>87.46–106.76 (pork);<br>92.30–116.51 (chicken)	LOD/LOQ:<br>Beef, Pork: 0.01/0.04<br>Chicken: 0.02/0.07	Jung et al. [59]
Bovine muscle, milk, milk powder (2 analytes)	Ethyl acetate extraction/ C18 dispersive clean-up	LC–MS/MS	0-0.75	89.27–100.65 (milk);<br>93.06–104.9 (milk powder);<br>91.00–106.15 (bovine)	LOD/LOQ:<br>milk: 0.102/0.339<br>milk powder: 0.066/0.22<br>bovine: 0.072/0.239	Rezende et al. [60]
Chicken muscle (4 analytes)	Acetonitrile + ammoniated water extraction followed by ethyl acetate/ Evaporation, ammoniated methanol dissolution, n-hexane defatting, C18 d-SPE	LC–MS/MS	0.075-0.9	98.3–108.1	CCα/ CCβ: 0.11/0.19<br>LOD/LOQ: 0.015/0.05	Imran et al. [61]

Chicken muscle and egg (12 analytes)	Derivatization, C18 and diatomaceous earth, hexane defatting/ Ethyl acetate extraction	UHPLC–MS/MS	0.1-1	92.7-108.7 (chicken); 90.9-97.3 (egg)	LOD/LOQ: 0.03/0.1	Zhang et al. (2017) [62]
Muscles (beef, pork, chicken)	Extraction ACN-H_2O (4:1, v/v), cleanup C18 and hexane saturated with ACN	LC-MS/MS	0.15-5	Bovine 99-100<br>Porcine 92-99<br>Chicken 98-100	LOD/LOQ: 0.05/0.15	This study

Table S8. Comparison with previously reported analytical methods for vancomycin

Matrix	Sample extraction and cleanup	Analytical technique	Calibration/linear range (μg/kg)	Recovery (%)	LOD/LOQ or CCα/ CCβ (μg/kg)	Reference
Animal tissues (muscle, liver, kidney) (6 analytes)	ACN-10% TCA (1:1, v/v) extraction/In-line MISPE clean-up	LC-MS/MS	0.5-200	68.1-90.5	LOD/LOQ: 4/12.5	Song et al. [67]
Milk	0.1% FA + ACN + TCA extraction / n-hexane + SMISPE clean-up	LC-MS/MS	1-100	83.3-92.1	LOD/LOQ: 0.5/1.0	Zhou et al. [68]
Milk (5 analytes)	EDTA-McIlvaine buffer + MeOH extraction/ HLB tandem MCX clean-up	UHPLC-MS/MS	1.0-20	93.0-102	LOD/LOQ: 0.33/1.0	Deng et al. [69]
Animal tissues (muscle, fat, liver, kidney)	40% ACN with 0.1% FA extraction/ n-hexane + SMISPE clean-up	LC-MS/MS	1-100	93.2-117	LOD/LOQ: 0.5/1.0	Chen et al. [70]
Fish meat	QuEChERS + water-ACN (1:9, v/v) with 0.1% FA extraction/ water-saturated n-hexane and 96 well SPE clean-up	UPLC-MS/MS	0.5-100	86.7-94.7	LOD/LOQ: 0.52/1.73	Shen et al. [71]
Fish tissues (muscle, liver, kidney)	ACN-water (7:3, v/v) with 0.1%FA extraction/ hexane saturated with ACN + PCX tandem C18 clean-up	LC-MS/MS	2-250	Muscle: 94.6-96.5<br>Liver: 80.2-83.6<br>Kidney: 75.5-79.8	LOD/LOQ:<br>Muscle: 2.0/5<br>Liver: 3.0/10<br>Kidney:3.0/10	Tang et al. [72]
Muscles (beef, pork, chicken)	15% ACN with ammonium acetate extraction/ Hexane saturated with water + MCX clean-up	LC-MS/MS	1-20	Bovine 100-106<br>Porcine 99-105<br>Chicken 98-103	LOD/LOQ: 0.3/1.0	This study

Table S9. Non-compliant findings for chloramphenicol reported in EU residue-monitoring programs during 2020–2023

	2020	2021	2022	2023
Targeted sampling	Lithuania: milk 6 (2.51%)<br>Poland: milk 1 (0.34%)<br>Germany: pork 3 (0.12%)<br>Spain: pork 1 (0.05%)	Germany: beef 1 (0.03%)<br>Netherlands: horse 1 (33.33%)<br>poultry 1 (0.20%)<br>Malta: milk 3 (1.63%)<br>Austria: pork 1 (0.07%)<br>Italy: pork 2 (0.28%)<br>Poland: poultry 2 (0.22%)	Czechia: pork 2 (1.10%)<br>Latvia: pork 1 (3.23%)<br>Netherlands: pork 1 (0.23%)<br>Poland: pork 1 (1.19%)<br>France: poultry 2 (0.19%)<br>Latvia: rabbits 1 (100%)	Czechia: beef 1 (1.02%)<br>Poland: milk 3 (0.88%)<br>Slovakia: milk 1 (3.33%)<br>Germany: pork 2 (0.13%)<br>Poland: pork 1 (0.16%)<br>Austria: poultry 2 (0.64%)<br>Germany: sheep/goats 1 (1.1%)
Suspect sampling	Germany: pork 11 (36.76%)	Malta: beef 1 (11.11%)		
Imported sampling				Denmark (from India): eggs 1 (100%)<br>Germany (from China): honey 1 (25%)<br>Greece (from Brazil): poultry 1 (100%)

Values in parentheses indicate the reported non-compliance rate.
